# LY86, LRG1 and PDE9A genes overexpression in umbilical cord blood hematopoietic stem progenitor cells by acute myeloid leukemia (M3) microvesicles

**DOI:** 10.1186/s40164-019-0147-8

**Published:** 2019-09-18

**Authors:** Farnaz Razmkhah, Sorayya Ghasemi, Masoud Soleimani, Sedigheh Amini Kafi-abad

**Affiliations:** 1 Hematology Research Center, Shiraz University of Medical Sciences, Khalili Street, Shiraz, Iran; 2 Cellular and Molecular Research Center, Basic Health Sciences Institute, Shahrekord University of Medical Sciences, Shahrekord, Iran; 3 Hematology Department, Faculty of Medicine, Tarbiat Modares University, Tehran, Iran; 4 Department of Pathology, Blood Transfusion Research Center, High Institute for Research and Education in Transfusion Medicine, Tehran, Iran; 5 Present Address: Department of Hematology and Blood Transfusion, School of Allied Medical Sciences, Iran University of Medical Sciences, Tehran, Iran

**Keywords:** Microvesicles, Hematopoietic stem progenitor cells, Leukemia, Cell communication

## Abstract

**Background:**

Microvesicles as a new device of cell–cell communication are potentially able to induce some phenotypes and genotypes of an origin cell in a target cell. We evaluate the role of leukemia microvesicles on the leukemia stem cells (LSCs)-specific genes expression in healthy hematopoietic stem progenitor cells (HSPCs).

**Methods:**

HL-60 and NB-4 cell lines were selected for microvesicles isolation by ultracentrifugation. Healthy HSPCs were obtained by magnetic association cell sorting (MACS) and CD-34 micro-beads from umbilical cord blood samples and then, were treated with 20 and 40 μg/ml leukemia microvesicles for 10 days, respectively. *LY86*, *LRG1* and *PDE9A* genes expression as LSC specific genes were analyzed by QRT-PCR. Surface CD-34 antigen as stemness marker was measured by flow cytometry technique.

**Results:**

Healthy HSPCs showed a significant increase in LSC specific genes expression after treatment with both 20 and 40 μg/ml leukemia microvesicles at day 10. All studied groups showed more than 70% surface CD-34 antigen at the last day of experiment which proved HSPCs stemness.

**Conclusion:**

Our results suggest that healthy HSPCs can be transformed genetically by leukemia microvesicles to over express LSC specific genes. This may be further evidence of leukemia-like transformation by leukemia microvesicles.

**To the respectful editor,**

Microvesicles, as units of biological information, play a remarkable role in cell–cell interactions [1]. Numerous studies have shown that microvesicles are able to phenotypically transform a target cell (normal or tumor cell) to express some proteins [2], mRNAs [3] and microRNAs [4] of origin cell. For example, genomic instability as one of the first events in cancer, can be induced in normal transplant by leukemia microvesicles [5, 6]. It seems that microvesicles are passing a crucial step to be known as the first factors in cancer initiation, progression and invasion.

Acute myeloid leukemia (AML) is created from genomic alterations in a healthy hematopoietic stem cell (HSC) which is now called leukemia stem cell (LSC). This new cell keeps the ability of self-renewal but is affected by cell death, proliferation and differentiation [7]. In the leukemia microenvironment, leukemia and healthy cells, are in regular cell–cell communication through microvesicles. This study tries to determine whether leukemia microvesicles are able to affect healthy HSPCs and dysregulate some genes such as *LY86*, *LRG1* and *PDE9A* as LSC specific genes which have fully different patterns of expression in HSC and LSC [8].

Isolated leukemia microvesicles from HL-60 and NB-4 cell lines were assessed by DLS technique (Additional file 1) and showed that all microvesicles are less than 1 μm in diameter which is the maximum limit of size for microvesicles (Additional file 2). Then healthy HSPCs from umbilical cord blood samples were treated with 20 and 40 μg/ml leukemia microvesicles for 10 days. CD-34 positive cells (98%) at day 0, still showed a high level of CD-34 antigen (more than 70%) as stemness marker after 10-day treatment with leukemia microvesicles (Additional file 3).

Quantitative Real Time PCR (Table 1) was performed at day 10 of experiment to evaluate any fold change of three selected genes expression in healthy HSPCs. *LY86* gene expression significantly increased (P < 0.001) with both doses of leukemia microvesicles (20 and 40 μg/ml) in both studied groups (treatment with NB-4 and HL-60 microvesicles) as shown in Fig. 1a. *LY86* gene overexpression in HSPCs treated with NB-4 microvesicles was more remarkable than in HSPCs treated with HL-60 microvesicles. This change is also dose dependent which is more distinct in HSPCs treated with 40 μg/ml than 20 μg/ml microvesicles.

**Table 1:** Primer sequences

Gene name	Primer type	Primer sequence
*LY86*	Forward	5-GAA GGA AAG GAG AGC AGA TTT AC-3
*LY86*	Reverse	5-TGA TAG TAG CAT TGG CAC AGG-3
*LRG1*	Forward	5-TCT TGG AGC AGA CAG CGA C-3
*LRG1*	Reverse	5-TTT CGG CAG GTG GTT GAC AG-3
*PDE9A*	Forward	5-ATA ACC ACA AGA AGT TGA CTC CTC-3
*PDE9A*	Reverse	5-GCA GCT CAG CAT CTC ATT GG-3
*HPRT1*	Forward	5-CCT GGC GTC GTG ATT AGT G-3
*HPRT1*	Reverse	5-TCA GTC CTG TCC ATA ATT AGT CC-3

**Fig. 1:**
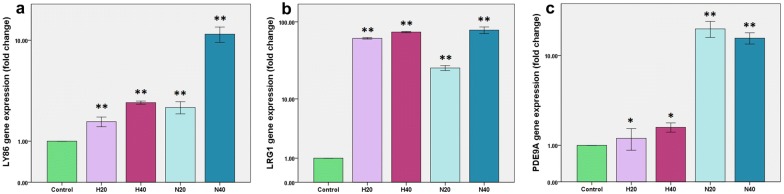
LY86, LRG1 and PDE9A genes expression in studied groups treated with HL-60 microvesicles (H20: 20 μg/ml microvesicles, H40: 40 μg/ml microvesicles) and NB-4 microvesicles (N20: 20 μg/ml microvesicles, N40: 40 μg/ml microvesicles). **a** LY86 gene fold change, **b** LRG1 gene fold change and **c** PDE9A gene fold change in different groups of HSPCs. *P < 0.05, **P < 0.001

The fold change of *LRG1* gene expression in HSPCs was extremely high in both studied groups (P < 0.001). This increase was also dose dependent which was more in HSPCs treated with 40 μg/ml than 20 μg/ml leukemia microvesicles (Fig. 1b).

*PDE9A* gene expression, however, did not change remarkably in HSPCs treated with HL-60 microvesicles, increased significantly at the last day of experiment (day 10) in a dose dependent manner (P < 0.05). This increase was more observable in HSPCs treated with NB-4 microvesicles (Fig. 1c) without a dose dependent manner (P < 0.001).

It seems that microvesicles are powerful enough to affect the pattern of gene expression in a target cell since Cardiomyocytes derived microvesicles/exosomes dysregulated 333 genes in fibroblast including 175 up regulations and 158 down regulations [9]. We previously showed the effect of leukemia microvesicles on survival and transformation of umbilical cord blood HSPCs [10–12]. Another study revealed that LSCs microvesicles are able to induce proliferation and migration of myeloid leukemia cells and support malignant cells [13]. In harmony with the above mentioned results, our study showed the ability of leukemia microvesicles in transformation of healthy HSPCs to over express LSC specific genes.

While leukemia microvesicles are able to transform a healthy HSPC through dysregulation of genes, mRNAs, microRNAs and proteins, there is always a question: will this transformation cause leukemia? This question is much more crucial when we know there is a close communication between leukemia and healthy cells in the bone marrow microenvironment through microvesicles. Therefore, any leukemia-like transformation of healthy HSPCs may be a step toward the leukemia, which may result in leukemia progression and relapse.

## Supplementary information

**Additional file 1.** Materials and methods.

**Additional file 2.** DLS technique. Isolated microvesicles were quantitatively size proved (80–1000 nm).

**Additional file 3.** CD34 analysis at the end day of experiment. All studied groups expressed more than 70% stemness marker.

## Data Availability

The datasets used and/or analyzed during the current study are available from the corresponding author on reasonable request.
