# Supplementary material for: LY86, LRG1 and PDE9A genes overexpression in umbilical cord blood hematopoietic stem progenitor cells by acute myeloid leukemia (M3) microvesicles
Source: Exp Hematol Oncol. 2019 Sep 18;8:23. doi: 10.1186/s40164-019-0147-8 (PMC6751795; doi:10.1186/s40164-019-0147-8)
Supplement: Supplementary file 1 — Additional file 1. Materials and methods. [file 40164_2019_147_MOESM1_ESM.docx]

**Methods**

*Cell preparation*

In this study, HL-60 and NB-4 cell lines were used as leukemia cell lines (both are acute promyelocytic leukemia) to provide leukemia microvesicles. They were cultured in RPMI 1640 medium containing 20% fetal bovine serum (FBS) (for HL-60 cell line) and 10% FBS (for NB-4 cell line), 100 U/ml Penicillin and 100 µg/ml Streptomycin at 37 °C, 5 % CO_2_ and full humidity.

*Microvesicle isolation and characterization*

After achieving enough leukemia cells, they were maintained in RPMI 1640 medium containing 0.6% bovine serum albumin (BSA), 100 U/ml Penicillin and 100 µg/ml Streptomycin at 37 °C, 5 % CO_2_ and full humidity overnight. Then, leukemia microvesicles were isolated and purified from cells supernatant by ultra-centrifuge ([13](#_ENREF_13)). Live and dead cells at 2,000 g, cell debris at 10,000 g and exosomes at 20,000 g were excluded from cells supernatant for final centrifugation at 20,000 g to achieve pure microvesicles. Their protein concentration was measured by Bradford assay to be used as microvesicles dose and their size was assessed by dynamic light scattering (DLS) technique to prove the isolation protocol.

*HSPCs sorting*

Cord blood bank of Iranian Blood Transfusion Organization (IBTO) provided cord blood samples in CPDA1 reagent after written consent form was obtained. Mononuclear cells (MNCs) were isolated by Lymphoprep and then magnetic-activated cell sorting (MACS) technique was used to sort HSPCs by CD-34 micro beads (Milteny Biotec, Auburn, CA) according to the manufacturer’s instruction.

*Treating HSPCs with leukemia microvesicles*

HSPCs were treated: 1- without any microvesicles (as control group), 2- with 20 and 40 ug/ml HL-60 microvesicles (as H-20 and H-40 groups), 3- with 20 and 40 ug/ml NB-4 microvesicles (as N-20 and N-40 groups), in 500 μl Stemline medium (Sigma-Aldrich) containing 50 ng/ml Thrombopoietin (TPO; Pepro Tech) and Fms-like tyrosine kinase 3 (FLT3; ORF Genetics) recombinant growth factors for 10 days.

*CD-34 analysis*

At the last day of experiment, cells were washed and stained by CD-34 antibody (PE-eBioscience, USA) for HSPC stemness evaluation through flow cytometry technique. Also, CD-34 analysis was performed at day 0 to evaluate the purity of sorted cells by MACS technique.

*QRT-PCR for genes expression*

After being treated with leukemia microvesicles, cells were washed to exclude any remained microvesicle. Total RNAs were extracted by RNX Plus reagent (Sinagen, Iran) and complementary DNAs (cDNAs) were then synthesized according to the manufacturer’s instructions (Fermentase). Quantitative real-time polymerase chain reaction (PCR) was performed to evaluate gene expression fold change according to ΔΔ*Ct* method using Applied Biosystems StepOne real-time system (Applied Biosystems, Foster City, CA, USA), SYBR Green PCR master mix (TaKaRa, Japan) and special primers (Table 1) which were normalized with *HPRT* gene as internal control.

*Statistical analysis*

Data from three different experiments were statistically analyzed using SPSS 22 (Microsoft, Chicago, IL, USA). One way ANOVA test was applied for comparing means among studied groups and Tukey test was done to find significant difference between groups. Adjusted level less than 0.05 was considered statistically significant.
