# Supplementary figures and images for: LY86, LRG1 and PDE9A genes overexpression in umbilical cord blood hematopoietic stem progenitor cells by acute myeloid leukemia (M3) microvesicles
Source: Exp Hematol Oncol. 2019 Sep 18;8:23. doi: 10.1186/s40164-019-0147-8 (PMC6751795; doi:10.1186/s40164-019-0147-8)

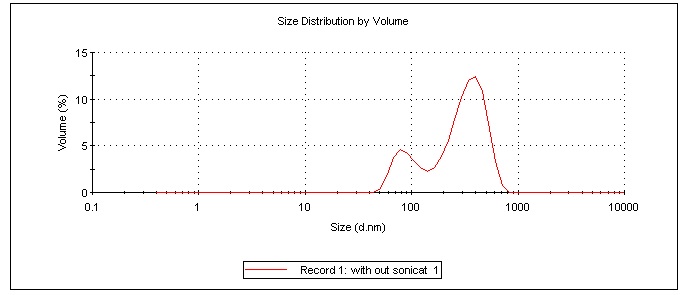

Supplement: Supplementary file 2 — Additional file 2. DLS technique. Isolated microvesicles were quantitatively size proved (80–1000 nm). [file 40164_2019_147_MOESM2_ESM.jpg]

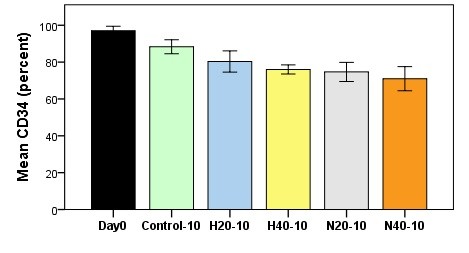

Supplement: Supplementary file 3 — Additional file 3. CD34 analysis at the end day of experiment. All studied groups expressed more than 70% stemness marker. [file 40164_2019_147_MOESM3_ESM.jpg]
